# Transcriptomic Profiling of Sugarcane White Leaf (SCWL) Canes during Maturation Phase

**DOI:** 10.3390/plants13111551

**Published:** 2024-06-04

**Authors:** Karan Lohmaneeratana, Kantinan Leetanasaksakul, Arinthip Thamchaipenet

**Affiliations:** 1 Department of Genetics, Faculty of Science, Kasetsart University, Bangkok 10900, Thailand; karan.l@ku.th; 2 National Center for Genetic Engineering and Biotechnology, National Science and Technology Development Agency, Pathumthani 12120, Thailand; kantinan.lee@biotec.or.th; 3 Omics Center for Agriculture, Bioresources, Food and Health, Kasetsart University (OmiKU), Bangkok 10900, Thailand

**Keywords:** sugarcane white leaf, phytoplasma, transcriptome, plant defense response, biotic stress

## Abstract

Sugarcane white leaf (SCWL) disease, caused by *Candidatus* Phytoplasma sacchari, results in the most damage to sugarcane plantations. Some SCWL canes can grow unnoticed through the maturation phase, subsequently resulting in an overall low sugar yield, or they can be used accidentally as seed canes. In this work, 12-month-old SCWL and asymptomatic canes growing in the same field were investigated. An abundance of phytoplasma in SCWL canes affected growth and sugar content as well as alterations of transcriptomic profiles corresponding to several pathways that responded to the infection. Suppression of photosynthesis, porphyrin and chlorophyll metabolism, coupled with an increase in the expression of chlorophyllase, contributed to the reduction in chlorophyll levels and photosynthesis. Blockage of sucrose transport plausibly occurred due to the expression of sugar transporters in leaves but suppression in stalks, resulting in low sugar content in canes. Increased expression of genes associated with MAPK cascades, plant hormone signaling transduction, callose plug formation, the phenylpropanoid pathway, and calcium cascades positively promoted defense mechanisms against phytoplasma colonization by an accumulation of lignin and calcium in response to plant immunity. Significant downregulation of *CPK* plausibly results in a reduction in antioxidant enzymes and likely facilitates pathogen invasion, while expression of sesquiterpene biosynthesis possibly attracts the insect vectors for transmission, thereby enabling the spread of phytoplasma. Moreover, downregulation of flavonoid biosynthesis potentially intensifies the symptoms of SCWL upon challenge by phytoplasma. These SCWL sugarcane transcriptomic profiles describe the first comprehensive sugarcane–phytoplasma interaction during the harvesting stage. Understanding molecular mechanisms will allow for sustainable management and the prevention of SCWL disease—a crucial benefit to the sugar industry.

## 1. Introduction

Sugarcane (*Saccharum* sp.), a grass monocot plant in the Family *Poaceae*, is one of the most important economic crops for the sugar industry as well as the production of ethanol, molasses, and animal feed. Thailand is the second-largest sugar exporter after Brazil and is ranked fourth for sugar production worldwide (US Department of Agriculture, 2023/2024). However, sugar production from cane has been seriously affected by several sugarcane diseases. One of the most destructive effects on sugar yield from canes in Thailand and other regions is sugarcane white leaf (SCWL) disease. The economic loss caused by SCWL disease on income from sugarcane cultivation was estimated to be around USD 30–40 million annually [1].

SCWL disease is caused by an obligate pathogenic bacterium that lacks a cell wall, *Candidatus* Phytoplasma sacchari, which so far has been impracticable to culture in laboratory conditions [2]. SCWL phytoplasma is transmitted via insect vectors, *Matsumuratettix hiroglyphicus* Matsumura [3] and *Yamatotettix flavovittatus* Matsumura [4]. After transmission, the bacterium generally localizes in phloem and uses metabolic pathways from the sugarcane host [5]. Based on 16S rRNA classification, SCWL phytoplasma is closely related to sugarcane grassy shoot (SCGS) and rice yellow dwarf (RYD) [6,7].

Symptoms of SCWL disease can be observed in every growth stage of sugarcane from seed canes or cane setts to the mature stage. In general, new emerging buds and shoots from the cane setts emerge with bushy slim white leaves of a soft texture that grow from slender chlorotic shoots [7]. Degrees of leaf chlorosis start from pale green to white with considerable tiller proliferation and stunting [8]. Variation in symptoms depends on the stage of sugarcane growth and development of disease. Although SCWL sugarcane is recognized and eliminated while it is young, less symptomatic canes can be missed visually and remain in the field as SCWL carriers. At the maturation phase (12 months old), when sugarcanes contain the highest sugar yield [9], such SCWL canes are harvested together with the healthy ones and result in a low yield of sugar production. Moreover, they could be unintentionally prepared as seed canes for the next propagation cycle that will quickly spread SCWL disease to other sugarcane plantations.

In Thailand, Khon Kaen 3 (KK3) is one of the most popular sugarcane cultivars for the sugar industry due to its high sugar productivity and vigorous growth in various soil conditions [10], but KK3 is not tolerant to SCWL disease. In this study, 12-month-old SCWL KK3, growing together with asymptomatic KK3 in the same field, were harvested and investigated. To determine the sugarcane genes that play important roles in SCWL pathogenesis, transcriptomic profiles of SCWL sugarcane leaves and stalks were undertaken as both organs perform the distinct metabolic functions of photosynthesis and sugar storage, respectively. Molecular mechanisms and related pathways of the sugarcane host affected by SCWL phytoplasma have been identified.

## 2. Results

### 2.1. Growth Characteristics and Phytoplasma Detection of SCWL Sugarcanes

Twelve-month-old SCWL sugarcanes showed symptoms of chlorosis leaf, 0.65 times smaller stalk diameter, 0.65 times shorter height, and 0.69 times lower sugar content than those of asymptomatic sugarcanes collected from the same plantation (Table 1, Figure S1A–C). To evaluate the numbers of active phytoplasma in leaves and stalks of sugarcane samples, the gene expression ratio of 16S/18S rRNA of phytoplasma/sugarcane was calculated. SCWL sugarcanes harbored 10^4^ times more phytoplasma than those of asymptomatic canes (Table 1). After propagation of the SCWL seed canes under greenhouse conditions for 2 months, newly emerged buds produced tillers and stunting with slim and narrow white leaves (Figure S1D).

### 2.2. RNA-Sequencing and Assembly

**Figure 1:**
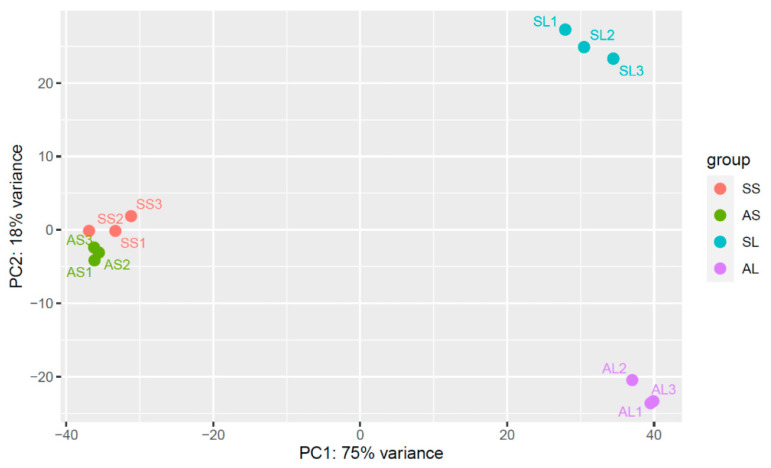
Principal component analysis (PCA) of gene expression from RNA-seq data of asymptomatic and symptomatic sugarcanes. AS, asymptomatic stalks; SS, symptomatic stalks; AL: asymptomatic leaves; SL, symptomatic leaves. The two principal components determine 93% of the total variance.

Total RNA-seq data of leaves and stalks of SCWL and asymptomatic sugarcanes were analyzed in triplicate (BioProject number PRJNA719388). More than 671 million raw reads (sequencing depth of 53.8–59.8 million of 150 bp paired-end read per library) were generated. About 647 million clean reads were filtered and 69.48% to 80.66% of the reads were mapped to the monoploid sugarcane genome reference (https://sugarcane-genome.cirad.fr; accessed on 14 February 2020) [11] (Table S1). Principal component analysis (PCA) of gene expression showed that SCWL and asymptomatic sugarcanes clustered separately in both leaves and stalks (Figure 1) and the volcano plot showed a reasonable distribution of gene expression (Figure S2). In leaves, 4799 and 1485 differentially expressed genes (DEGs; *p*-value < 0.05, log_2_ FC > 1) were upregulated and downregulated, respectively, while in stalks, 994 and 743 DEGs were upregulated and downregulated, respectively (Table 2). Co-expression of DEGs between leaves and stalks contained 330 upregulated and 92 downregulated DEGs, respectively (Table 2).

### 2.3. Gene Ontology (GO) Annotation and KEGG Pathway-Enrichment Analysis

The significant upregulated and downregulated DEGs of leaves and stalks were annotated with GO terms (Table 2). In leaves, DEGs were categorized and enriched in biological processes (e.g., photosynthesis, carbohydrate metabolic processes, generation of precursor metabolites and energy, cellular amino acid and protein metabolic processes), cellular component (membrane and thylakoid), and molecular function (e.g., transporter activity, catalytic activity, kinase/transferase activity, carbohydrate binding, and transcription factor activity) (Figure S3A), while in stalks, significant DEGs were categorized in biological processes (e.g., regulation of metabolic process, macromolecule modification, regulation of gene expression, protein modification processes, and regulation of biological processes) and molecular function (e.g., transcription factor activity, transferase activity, kinase activity, and transporter activity) (Figure S3B).

**Figure 2:**
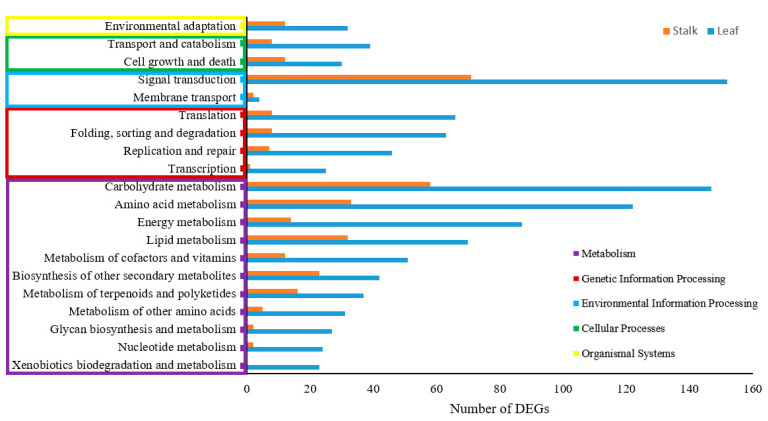
Number of DEGs in each category of KEGG pathways in SCWL leaves and stalks.

**Figure 3:**
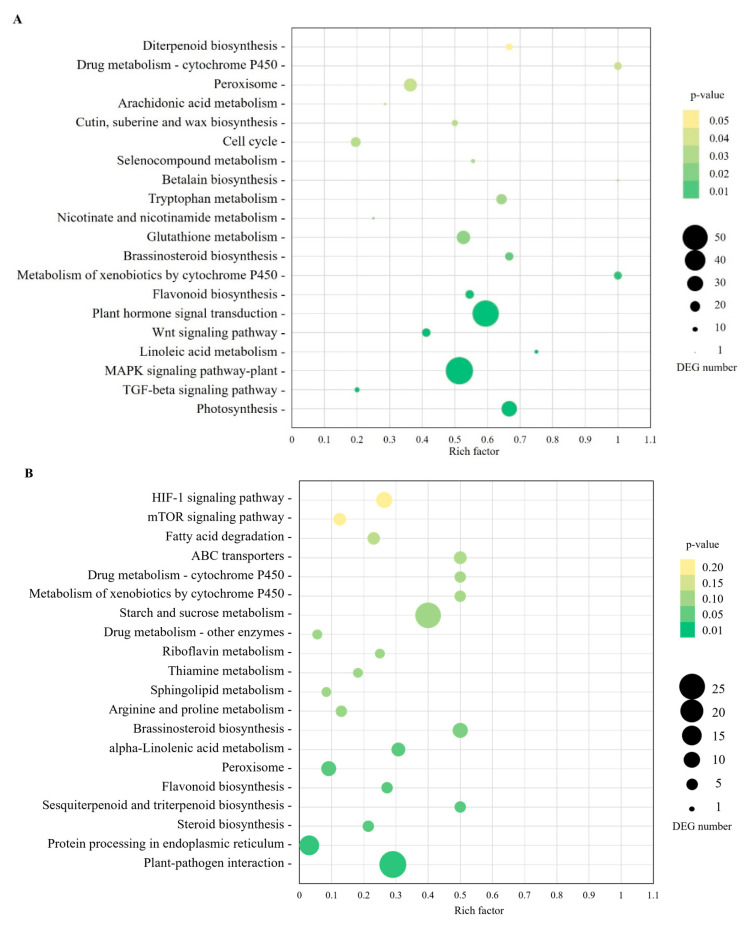
Top 20 KEGG pathway enrichment of DEGs between SCWL and asymptomatic sugarcanes in leaves (**A**) and stalks (**B**). The rich factor is a ratio of the number of DEGs and the total genes. The size and color of the bubble represent the number of DEGs and the *p*-value, respectively.

As a result of the KEGG analysis, upregulated and downregulated DEGs of leaves and stalks were annotated for functionality (Table 2). The most frequent pathways were metabolism, genetic information processing, environmental information processing, cellular processes, and organismal systems (Figure 2). To attain pathway enrichment, the ‘piano in R’ package was used. The enriched DEGs in leaves were associated with various functions including photosynthesis, signal transduction, flavonoid biosynthesis, metabolism of terpenoids and polyketides (Figure 3, Table S2). The most enriched upregulated pathways included mitogen-activated protein kinase (MAPK) signaling pathways, monoterpenoid biosynthesis, and plant–pathogen interactions, while the most enriched downregulated pathways were photosynthesis, photosynthesis antenna proteins, porphyrin and chlorophyll metabolism, signal transduction, carotenoid biosynthesis, and carbon fixation in photosynthetic organisms (Table S2). In stalks, the enriched DEGs included plant–pathogen interactions and protein processing in the endoplasmic reticulum pathway (Figure 3, Table S3). The most enriched upregulated pathways encompassed brassinosteroid biosynthesis, sesquiterpenoid and triterpenoid biosynthesis, while the most enriched downregulated pathways were starch and sucrose metabolism, glycolysis/gluconeogenesis, diterpenoid biosynthesis, flavonoid biosynthesis and carbon fixation in photosynthetic organisms (Table S3).

### 2.4. Phytoplasma-Affected Metabolic Processes of Sugarcane

From GO and KEGG pathway analyses, infection by phytoplasma evidently influenced the expression of many genes related to metabolic pathways in leaves and stalks including chlorophyll metabolism and photosynthesis, sucrose accumulation, plant–pathogen interactions, plant hormone signaling transduction, and secondary metabolites.

#### 2.4.1. Chlorophyll Metabolism and Photosynthesis

**Figure 4:**
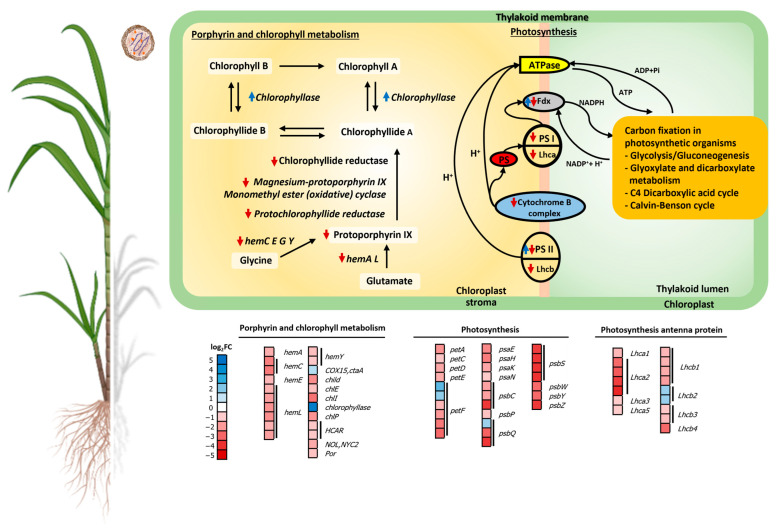
Effect of phytoplasma on photosynthesis and chlorophyll metabolism in SCWL sugarcane. Heatmaps of upregulated and downregulated DEGs of porphyrin and chlorophyll metabolism, photosynthesis, and photosynthesis antenna proteins are shown. Blue arrow, upregulation; red arrow, downregulation.

In SCWL leaves, phytoplasma significantly suppressed the photosynthetic pathway and chlorophyll content, resulting in an alteration of the downstream pathways. Most DEGs of porphyrin and chlorophyll metabolism were downregulated including protoporphyrin IX-related genes; chlorophyllide b reductase (*NYC1*, K13606), geranylgeranyl diphosphate (*chlP*, K10960), 7-hydroxymethyl chlorophyll a reductase (*HCAR*, K18010), magnesium chelatases (*chlH*, K03404; K03405), magnesium-protoporphyrin IX monomethyl ester cyclase (*chlE*, K04035), protochlorophyllide reductase (*por*, K00218); and heme-related gene clusters [*hemA* (K02492), *hemC* (K01749), *hemE* (K01599), *hemL* (K01845), and *hemY* (K00231)]; while chlorophyllase (K08099) and cytochrome c oxidase assembly protein subunit 15 (*COX15*, K02259) were upregulated (Figure 4 and Table S4).

Meanwhile, most DEGs of photosynthesis were downregulated including cytochrome b6/f complex subunits [*PetA*, K02634; *PetC*, *PetD*, (K02636–7)], photosystem I subunits (*PsaE*, K02693; *PsaH*, K02695; *PsaK*, K02698; *PsaN*, K02701), photosystem II Psb proteins [*PsbC*, K02705; *PsbP*, K02717; *PsbS*, K03542; *PsbW*, K02721; *PsbY, PsbZ*, (K02723–4)], and photosynthetic electron transport, i.e., plastocyanin and ferredoxin [*PetE*, *PetF* (K02638–9)]. Moreover, the DEGs of photosynthesis antenna protein were also downregulated including the light-harvesting chlorophyll protein complex [*Lhca1*, *Lhca2*, *Lhca3*, (K08907–9); *Lhca5*, *Lhcb1*, *Lhcb2*, *Lhcb3*, *Lhcb4* (K08911–5) (Figure 4 and Table S4).

#### 2.4.2. Regulation of Genes Related to Sucrose Accumulation

**Figure 5:**
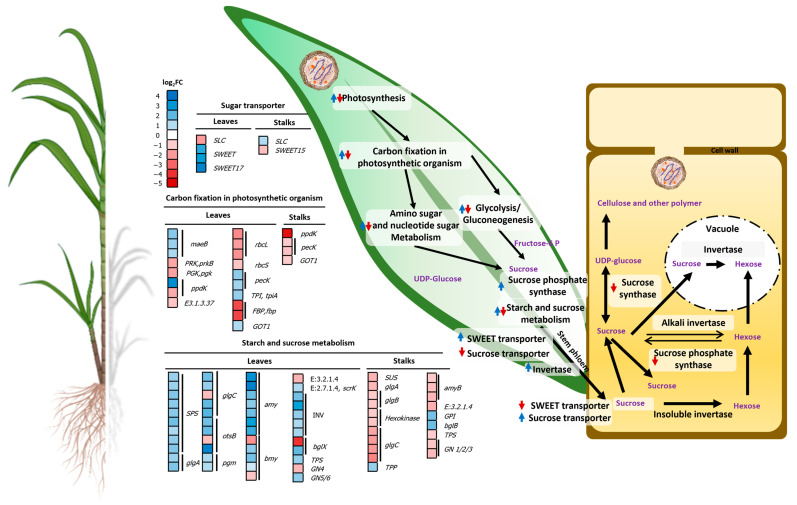
Effect of phytoplasma on sucrose accumulation in SCWL leaves and stalks. Heatmaps of upregulated and downregulated DEGs of sugar transporters, carbon fixation in photosynthetic organisms, starch and sucrose metabolism are shown. Blue arrow, upregulation; red arrow, downregulation.

The accumulation of sucrose was negatively affected in sugarcanes infected by phytoplasma. In leaves, transcripts related to sucrose accumulation were altered by most DEGs of carbon fixation in photosynthetic organisms including the upregulation of aspartate aminotransferase (*GOT1*, K14454), phosphoenolpyruvate carboxykinase (*pecK*, K01610), malate dehydrogenase (*maeB*, K00029); and downregulation of fructose-1,6-biphosphatase I (*FBP*, K03841), pyruvate phosphate dikinase (*ppdK*, K01006), and ribulose-bisphosphate carboxylases (*rubisco*, K01601–2) (Figure 5 and Table S4). Forty-three DEGs of starch and sucrose metabolism were upregulated including alpha-amylase (*amy*, K01176), beta-amylase (*bmy*, K01177), beta-glucosidase (*bglX*, K05349), invertase (*sacA*, K01193), starch synthase (*glgA*, K00703), sucrose phosphate synthase (*SPS*, K00696), trehalose 6-phosphate phosphatase (*otsB*, K01087), and trehalose 6-phosphate synthase (*TPS*, K16055). Additionally, SWEET transporters (*SWEET*, *SWEET17*; K15382) were highly upregulated but the H^+^/sugar cotransporter (SUT) (*SLC*, K15378) was downregulated (Figure 5 and Table S4).

In stalks, carbon fixation in photosynthetic organisms was downregulated including *GOT1*, *pecK*, and *ppdK*. Seventeen DEGs of starch and sucrose metabolism were also suppressed including *bmy*, *glgA*, *glgC*, sucrose synthase (*SUS*, K00695), and *TPS*, whereas beta-glucosidase (K05350), glucose-6-phosphate isomerase (K01810), and *otsB* were upregulated (Figure 5 and Table S5). On the contrary to leaves, SWEET transporter, *SWEET15* (K15382), was downregulated but *SLC* was upregulated (Figure 5 and Table S5).

#### 2.4.3. Regulation of Genes Related to Plant–Pathogen Interactions

**Figure 6:**
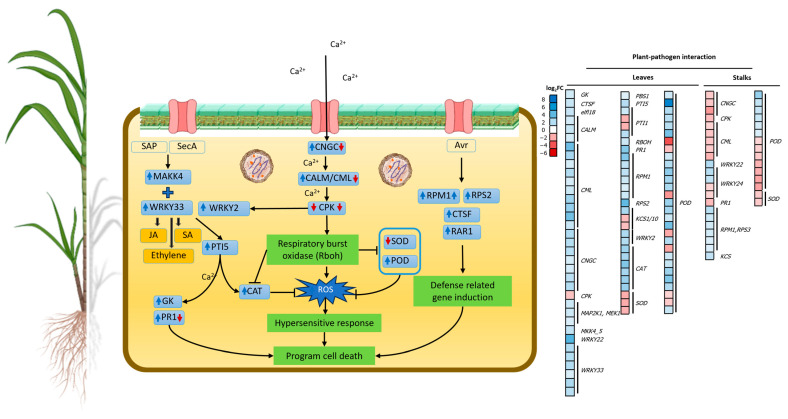
Effect of phytoplasma on plant–pathogen interaction in SCWL sugarcane leaves and stalks. Heatmaps of upregulated and downregulated DEGs of the plant–pathogen interaction are shown. Blue arrow, upregulation; red arrow, downregulation.

Most DEGs in plant–pathogen interaction pathways were upregulated (Figure 6 and Table S4) including calmodulin (*CALM*, K02183), cathepsin F (*CTSF*, K01373), cyclic nucleotide-gated channel (*CNGC*, K05391), disease resistance proteins [*RAR1* (K13458), *RPM1* (K13457), and *RPS2* (K13459)], glycerol kinase (*GK*, K00864), mitogen-activated protein kinase kinase (*MAP2K1*, K04368; *MKK4/5*, K13413), MAP kinase substrate 1 (*MKS1*, K20725), and pathogenesis-related genes including transcriptional activator pattern-triggered immunity (*PTI5*, K13433), pathogenesis-related protein (*PR1*, K13449), and WRKY transcription factors (*WRKY2*, K18835; *WRKY22*, K13425; *WRKY33*, K13424). Downregulated DEGs included calcium-dependent protein kinase (*CPK*, K13412), elongation factor Tu (*tuf*, K02358), and 3-ketoacyl-CoA synthase 11 (*KCS*, K15397). In addition, one of the plant defense-related genes, invertase (K01193), was accelerated during sugarcane–phytoplasma interactions along with callose synthases (K11000) (Table S4). In stalks, upregulated DEGs included *RPM1*, *KCS*, *WRKY22*, *WRKY24*, and *WRKY33*, while *CALM*, *CNGC*, *CPK10*, and *PR1* were downregulated (Table S5).

KEGG pathway analysis of SCWL leaves revealed 113 DEGs encoding transcription factors (TFs) which can be categorized into six types (Table S6). The largest category was helix-turn-helix proteins including MYB (*MYBP*, K09422), and MYB-related TF LHY (*LHY*, K12133). MYB TFs were upregulated along with other TFs including TGA (*TGA*, K14431), phytochrome-interacting factor 4 (*PIF4*, K16189), teosinte branched1/cycloidea/proliferating cell factors (*TCP*, K16221), and WRKYs (Table S6). In stalks, TFs such as *MYB*, *TGA*, *WRKY22*, and *WRKY33* were upregulated, while *PIF4* was downregulated (Table S7).

#### 2.4.4. Regulation of Genes Related to Signal Transduction by Plant Hormones

In SCWL leaves, most DEGs of the plant hormone signal transduction pathway were altered (Table S4), including upregulated DEGs of abscisic acid (ABA) [e.g., ABA receptor PYR/PYL family (*PYL*, K14496), ABA responsive element binding factor (*ABF*, K14432), catalase (*CAT*, K03781), and MAPK kinase kinase (*MAPKKK17/18*, K20716)], ethylene (ETH) signaling pathway [e.g., ethylene receptor (*ETR*, K14509), ETH-responsive TF 1 (*ERF1*, K14516), endochitinase B (*ChiB*, K20547), and MAPK kinase (*MKK4/5*, K13413; *MKK9*, K20604)], auxin (AUX) (*AUX1*, K13946; *AUX/IAA*, K14484; *GH3*, K14487; *SAUR*, K14488), cytokinin (CK) (*A-ARR*, K14492; *AHP*, K14490; *HK*, K14489), gibberellin (GA) (*RGA*, K14494 and *PIF4*), jasmonic acid (JA) (*COI1*, K13463; *JAZ*, K13464), salicylic acid (SA) [*PR1*, K13449 and TGA TF (K14431)], and brassinosteroid (BR) (*TCH4*, K14504). On the contrary, *A-ARR* of CK, *PIF4* of GA, and *PR1* of SA in stalks were suppressed, whereas *JAR1* and *JAZ* of JA and *NPR1* of SA were upregulated (Table S5).

#### 2.4.5. Regulation of Genes Related to Secondary Metabolites

In leaves, key genes in flavonoid biosynthesis were downregulated (Table S4), including chalcone synthase (*CHS*, K00660), caffeoyl-CoA O-methyltransferase (*CCoAOMT*, K00588), chalcone isomerases (*CHIL*, K01859), flavonoid 3,5-hydroxylase (*CYP75A*, K13083), and flavonoid 3-monooxygenase (*CYP75B1*, K05280), while some were also downregulated in stalks (Table S5). Genes in phenylpropanoid biosynthesis were upregulated in SCWL leaves (Table S4), including cinnamyl-alcohol dehydrogenase (*CAD*, K00083), cinnamoyl-CoA reductase (*CCR*, K09753), coniferyl-aldehyde dehydrogenase (*REF1*, K12355), peroxidase (*POD*, K00430), phenylalanine ammonia-lyase (*PAL*, K10755), and shikimate O-hydroxycinnamoyltransferase (*HCT*, K13065), while some were found to be upregulated in stalks (Table S5).

In leaves, the DEGs of BR biosynthesis were upregulated (Table S4), including typhasterol/6-deoxotyphasterol 2-alpha-hydroxylase (*CYP92A6*, K20623), PHYB activation-tagged suppressor 1 (*CYP734A1*, K15639), steroid 22-alpha-hydroxylase (*DWF4*, K09587), and steroid 5-alpha-reductase (*DET2*, K09591), while some were also upregulated in stalks (Table S5). In SCWL leaves, the DEGs of diterpenoid biosynthesis were upregulated (Table S4), including ent-kaurene synthase (*GA2*, K04121), ent-kaurenoic acid monooxygenase (*KAO*, K04123), GA 2-beta-dioxygenase (*GA2ox*, K04125), and GA-44 dioxygenase (*GA20ox*, K05282), whereas some from stalks were downregulated including ent-kaurene oxidase (*GA3*, K04122), *GA2ox* and *GA20ox* (Table S5). Monoterpenoid, sesquiterpenoid, and triterpenoid biosynthetic genes including germacrene D synthase (K15803) and NAD+-dependent farnesol dehydrogenase (*FLDH*, K15891) were upregulated in both leaves and stalks (Tables S4 and S5).

In leaves, the DEGs of carotenoid biosynthesis including abscisic-aldehyde oxidase (*AAO*, K09842), abscisic acid 8′-hydroxylase 2 (*CYP707A*, K09843), β-carotene 3-hydroxylase (*crtZ*, K15746), carotenoid epsilon hydroxylase (*LUT1*, K09837), 9-cis-epoxycarotenoid dioxygenase (*NCED*, K09840), and xanthoxin dehydrogenase (K09841) were downregulated but abscisic-aldehyde oxidase was upregulated (Table S4). In stalks, *AAO* was upregulated, while β-carotene isomerase and *NCED* were downregulated (Table S5).

### 2.5. Verification of DEGs by Real-Time PCR

**Figure 7:**
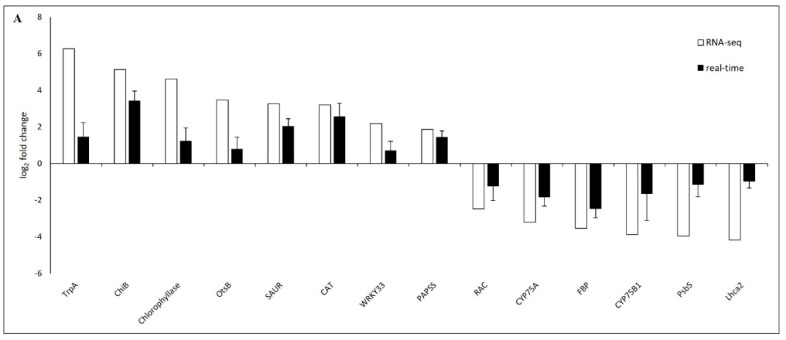

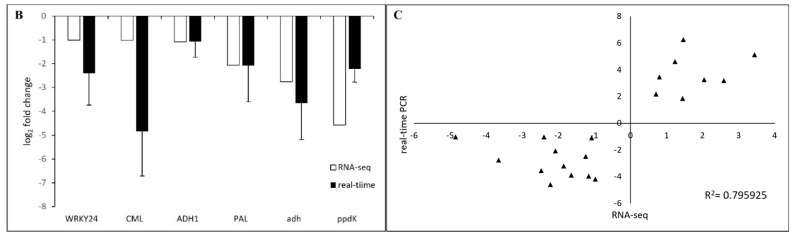
Validation and comparison of randomly selected DEGs of RNA-seq data (white bar) and real-time PCR quantification (black bar) of leaves (**A**), stalks (**B**) and correlation coefficients (*R*^2^) between RNA-seq and real-time PCR results for differential gene expression in leaves and stalks of asymptomatic and symptomatic sugarcane (**C**).

To validate RNA-seq data, a total of twenty randomly selected candidate genes from leaves and stalks with upregulation and downregulation (Table S8) were verified by real-time PCR. A comparison of RNA-seq with real-time PCR showed a correlation coefficient (*R^2^*) of 0.795925. The results confirmed the expression trends of all candidate genes in accordance with the analysis of the transcriptome data (Figure 7).

## 3. Discussion

The pathogenicity of SCWL phytoplasma for sugarcane is still unclear. Since phytoplasmas are difficult to culture in laboratory conditions and in vitro infection is exceptionally impracticable [2], 12-month-old SCWL and asymptomatic *Saccharum* hybrid cv. KK3 were collected from the same field and used for investigation to simulate the natural condition. The maturation phase is an important stage for harvesting since it gives the highest sugar yield with an economically acceptable marginal return for the sugar industry [9]. The phenotypic characteristics of SCWL sugarcanes including height, stalk diameter, and sugar content were significantly lower, which corresponded to a 10^4^ times higher phytoplasma expression ratio detected than those of asymptomatic canes. Furthermore, new buds emerging from such SCWL seed canes generated SCWL symptoms of a white grassy shoot phenotype with chlorosis leaves [6].

### 3.1. SCWL Phytoplasma Affects Chlorophyll Metabolism and Photosynthesis

In SCWL leaves, *NYC1* and *HCAR* that, respectively, reduce chlorophyll *b* (Chl *b*) and chlorophyll *a* (Chl *a*) in chlorophyll metabolism [12,13] were repressed similarly to that reported in grapevine leaves infected with Flavescence dorée (FD) phytoplasma [14]. Moreover, *hemA*, *hemC*, *hemE*, *hemL*, and *hemY* involved in the formation of protoporphyrin IX, a key precursor of chlorophyll biosynthesis, were downregulated along with *chlH*, *chlE* and *por* (Figure 4) that modify Mg^2+^ branching and form an isocyclic pentanone ring of chlorophyll [13,15]. High expression of chlorophyllase additionally degrades chlorophyll by cleaving the phytol tail and removing magnesium from the porphyrin ring [16,17]. Such downregulated mechanisms of porphyrin and chlorophyll gene expression together with the enhancement of chlorophyllase activity in leaves of SCWL sugarcane evidently explain the symptoms of leaf chlorosis and low chlorophyll content caused by SCWL phytoplasma. The findings agree with the growth limitation of phytoplasma-infected sesame plants where photosynthetic rates were reduced due to the depletion of chlorophyll content, photosystem II photoprotection, and photosynthetic capacity [18,19].

In this work, photosynthesis-related genes were mostly downregulated in SCWL leaves, which agrees with the observation for phytoplasma-infected Chinese jujube leaves in which the *Lhcb* gene family and photosystem II were downregulated [20,21]. Such suppression was also found in genes associated with photosystem I and II, including the cytochrome b6/f complex and ATP synthase in phytoplasma-infected grapevine [22,23], periwinkle plant [24], and coconut palm [25]. As part of defense mechanisms in response to biotic stress, photosynthesis genes are globally downregulated [26], which decrease phloem loading and carbohydrate accumulation of the infected leaves as a consequence [23] (Figure 4 and Figure 5).

The other downregulated pathway affecting photosynthesis, photoprotection, and signaling in SCWL sugarcanes was the carotenoid pathway. A reduction in carotenoid content was found in phytoplasma-infected apple, Chinese jujube, cranberry, grapevine, and Napier grass [14,20,27,28,29]. Low levels of the antioxidant carotenoids in phytoplasma-infected plants have a profound effect on plant physiology and response to stress since carotenoids play important roles in protecting cell damage caused by ROS [30], and protect chlorophyll and the photosynthetic apparatus from photodamage [31]. Moreover, carotenoids are precursors for plant hormone ABA; they are involved in the stress response [32] and work together with chlorophyll in the process of light harvesting during photosynthesis [33]. Therefore, downregulation of carotenoid-related genes in SCWL sugarcanes must have a downstream effect on ABA levels, which impacts the ability of the plant to respond to the phytoplasma and reduces the efficiency of photosynthesis.

### 3.2. SCWL Phytoplasma Alters the Gene Expression of Sucrose Accumulation

In SCWL leaves, several genes related to carbon fixation in photosynthetic organisms, such as *GOT1*, *maeB*, and *pecK*, were upregulated (Figure 5) similarly to those in leaves of cranberry infected with phytoplasmas [29], *Paulownia fortunei* [34], pepper, and *Ziziphus jujuba* Mill [35]. Moreover, *ppdK* that converts pyruvate to phosphoenolpyruvate (PEP) in the C4 photosynthesis pathway [36] was negatively regulated in SCWL leaves similar to that of phytoplasma-infected Paulownia [37]. In addition, *rubisco* was also downregulated like that of phytoplasma-infected Chinese jujube, indicating the limitation of photosynthesis imposed by the carboxylation [20].

In this study, the accumulation of sugar was negatively affected by SCWL phytoplasma by the upregulation of genes involved in starch and sucrose metabolism of leaves (source organ) such as alpha-amylase, beta-amylase, invertase, starch synthase, *SPS* and *TPS* (Figure 5), similarly to those of phytoplasma-infected Chinese jujube [21], grapevine [22], and tomato [38]. Such high expression of invertase, *SPS*, and *TPS* in SCWL leaves, but downregulation in stalks, suggests that sucrose transport is blocked and leads to a high sucrose content in leaves but low in stalks (the sink organ) [38]. Furthermore, such a block would affect sugar signaling and auxin crosstalk that play important roles in plant growth and development [39] and result in thinner stalks compared to the asymptomatic sugarcanes. Likewise, a sugar transporter, of the SWEET family, that maintains the equilibrium between membranes [40] was upregulated in SCWL leaves but downregulated in stalks (Figure 5), which would lead to unbalanced carbohydrate content between source and sink organs, similarly to those reported in coconut palm [41], periwinkle, tobacco [42], and tomato infected by phytoplasmas [43]. On the contrary, SUT that transports sucrose from the photosynthetic organ to the sieve element–companion cell complex was downregulated in SCWL leaves (Figure 5), which is similar to phytoplasma-infected tomato and plants expressing *SUT* antisense RNA, in which the rate of sucrose exudation was lower in the infected plants [43]. Moreover, phytoplasma possibly acts as an additional ‘sink’ to accumulate carbohydrates from photosynthesis and then blocks sugar transport from the leaf source to stalk sink [41]. Altogether, the loading of sugar from leaves to stalks was disrupted, which resulted in lower sugar content in SCWL stalks than that of asymptomatic sugarcanes.

### 3.3. SCWL Phytoplasma Alters the Expression of Genes Related to Plant–Pathogen Interactions

Sugars are not only the primary component furnishing energy but also a structural material for defense responses in plants [44]. Pathogens utilize sugar for their survival, while plants adapt their sugar production to stimulate plant defense responses. Invertases are one of the sucrose hydrolytic enzymes for carbohydrate degradation [45], and a key regulator for sucrose accumulation in sugarcane stalks [46]. *SUS* that was upregulated in SCWL stalks is localized in both companion cells and sieve elements of phloem and supplies UDP glucose to induce callose plugs in the sieve pores [47]. Such obstruction of the sieve tubes by callose deposition was previously reported in phytoplasma-infected *C. roseus*, *Euphorbia pulcherrina* [48], and *Vitis vinifera* L. cv. Chardonnay [23]. Moreover, invertase and callose synthases of SCWL sugarcanes were elevated in leaves, which may lead to sieve-tube occlusion, which is required to prevent the colonization of SCWL phytoplasmas. The results of this work are in accordance with those of phytoplasma-infected apple [49] and grapevine [23].

Calcium-binding proteins such as calmodulin (CAM) and calcium-dependent protein kinase (CPK) are involved in the signaling network in plant innate immunity [50]. CPK-CNGCs are cation transport channels regulated by CAM [51]. Those genes were upregulated in phytoplasma-infected American cranberry [29], *P. fortunei* [52], and Mexican lime [53], similarly to the results for SCWL sugarcanes. Upregulation of calcium signaling cascades sensed by a high expression of *CALM* in SCWL sugarcanes leads to Ca^2+^ accumulation. This accumulation should induce the expression of *CPK*, followed by *rboh*, which is a main source for ROS and plays an important role in the plant disease response [54]. Upregulation of *rboh* in SCWL sugarcanes implies that the pathogenic development of SCWL phytoplasma possibly required host Rboh to induce ROS and cell death, which resulted in the anchoring of SCWL disease. However, *CPK* expression in SCWL canes at the maturation phase was unexpectedly downregulated. The result would lead to a decrease in the activity of SOD, POD, and CAT, correlating with the silencing *CPK* in powdery mildew-infected wheat [55] and *CPK* mutants of Arabidopsis that exhibited vigorous pathogen growth and development of disease symptoms [56]. Therefore, downregulation of *CPK* may be a mechanism whereby SCWL phytoplasma triggers sugarcane to allow for more invasions of the pathogen.

MAPK cascades play important roles in the regulation of innate immune responses in plants [57] and directly control gene expression by phosphorylating TFs [58]. These genes were highly expressed in SCWL sugarcanes, indicating that infection by phytoplasma accelerated the expression of the MAPK signaling pathway and boosted the stress response in sugarcane. Furthermore, plant disease resistance genes involved in the innate immune response against pathogen invasion such as *RAR1*, *RPS2*, and *PTI5* [59] were upregulated in SCWL leaves, similarly to those found in witches’ broom disease (WBD) in *Nerium indicum* Mill [60]. PTI5, a transcriptional activator of ETH-responsive element binding protein, was enriched in SCWL sugarcanes, which potentially induces the expression of *CAT* [61]. In concordance with our results, *PR1* encoding a toxic protein against pathogen invasion [62] was upregulated in periwinkle leaf yellowing (PLY) phytoplasma-infected periwinkle [63], *Ca.* P. solani-infected tomato [30], *Ca.* P. asteris-infected Arabidopsis [64], *Ca.* P. mali-infected apple [65], and *Ca.* P. solani-infected grapevine [66]. Acceleration of *GK* in SCWL sugarcanes agrees with that of WBD in *Nerium indicum L*., which is involved in phytoplasma resistance [67]. In this work, WRKY22 that modulates ETH, JA, and SA signaling [68] was upregulated in SCWL sugarcanes. Upregulation of WRKY33 in SCWL sugarcanes is similar to that of *Xanthomonas albilineans*-infected sugarcanes [69]. Moreover, upregulation of *PTI5* together with *WRKYs* in SCWL sugarcanes may activate the defense-related genes involved in phytoalexin production, which plays a crucial role in plant defense against pathogens and contributes to the overall resistance of the plant [70]. Notably, some DEGs were upregulated in leaves but downregulated in stalks due to the fact that leaves are frequently exposed to pathogens and promptly activate defense mechanisms to prevent the invasion [71], whereas stalks primarily serve as structural and nutrient transport rather than having a defense function [72].

### 3.4. SCWL Phytoplasma Accelerates Plant Hormone Signaling Transduction

Genes responsible for plant hormone signaling pathways were mostly upregulated in SCWL sugarcanes including ABA, AUX, BR, CK, ETH, GA, JA, and SA, which are involved in plant defense responses, particularly host–pathogen interactions [73]. Similar to these results, ABA signaling pathway-related genes such as *PYL*, *ABF*, and *NCED* of phytoplasma-infected apple, Mexican lime, mulberry, and *Paulownia fortunei* were upregulated [53]. Genes involved in the AUX biosynthesis of phytoplasma-infected Mexican lime [53] and sesame [32] were upregulated along with CK and GA-related genes, which are similar to those of SCWL sugarcanes in this work. Furthermore, SCWL phytoplasma caused the upregulation of genes involved in the ETH signaling pathway such as *ERF* and *ETR*, similar to those of *Ca.* P. solani-infected grapevine [74], *Ca.* P. aurantifolia-infected Mexican lime [53], and *Paulownia* witches’ broom phytoplasma-infected *P. fortunei* [75]. Upregulation of ETH genes is associated with the activation of genes involved in the production of ROS, which are important components of the plant defense response [22].

Phytoplasma infection controls cell balance and plant development by secretion of the SAP11 effector that targets TCP TF, which, in turn, controls cell proliferation, cell maturation, plant development, and senescence by binding to the lipoxygenase (*LOX*) promoter in *Ca.* P. asteris AY-WB-infected Arabidopsis [76] and mediates the production of oxylipin, a precursor of JA biosynthesis [77]. TCP5 binds directly to the promoter of the *PIF4* gene to increase its expression level and interacts with the PIF4 protein [78]. In tomato and grapevine infected with *Ca.* P. solani, *LOX* was upregulated [30,79]. In this work, TCPs were upregulated in SCWL leaves and resulted in an enhancement of the expression of *LOX***,** which may lead to increased levels of JA in sugarcanes. Accumulation of JA can in turn trigger the expression of the downstream JA-responsive genes involved in the plant defense against SCWL phytoplasma. Upregulation of such genes in ‘*Ca*. P. asteris’ strain AY-WB-infected Arabidopsis was similarly observed [76].

The expression of several SA-related genes such as *PR1* was upregulated in SCWL sugarcane, similarly to those of phytoplasma-infected apple, Arabidopsis, grapevine, Madagascar periwinkle, and tomato [30,64,65,80,81]. The PR1 regulates gene expression by interacting with TGA TF that interacts with the promoter of *PR* itself in the presence of SA [82]. Thus, the induction of *PR1* expression would promote the biosynthesis of the plant SA [83]. Upregulation of *TCH4* in SCWL sugarcane is similar to that of phytoplasma-infected paulownia [84], which is likely to be involved in the biosynthesis of hemicellulose, a complex carbohydrate component of plant cell walls [85]. Together with the downregulation of *BRI1*, these results suggest a potential plant defense mechanism by sugarcanes against SCWL phytoplasma.

### 3.5. SCWL Phytoplasma Alter the Expression of Genes Involved in Secondary Metabolites

Phenylpropanoid precursors including benzenoids, coumarins, flavonoids, hydroxycinnamates, and lignin are involved in plant development and plant–pathogen interactions relating to environmental stress and disease tolerance [86]. In this study, *CAD*, *CCR*, *4CL*, *PAL*, and *POD* (which play roles in the phenylpropanoid pathway) were upregulated in SCWL sugarcanes, leading to an increase in lignin content [87] that would be related to strengthening the cell walls of sugarcane in response to an attack by phytoplasma. Furthermore, several upregulated MYB TFs that are responsible for the regulation of biosynthetic genes for phenylpropanoid and lignin were detected in SCWL sugarcanes like those reported in Arabidopsis [88]. Therefore, the contents of various phenylpropanoid compounds including lignin, flavonoids, and coumarins, which play crucial roles in the resistance against phytoplasma infection [89], were potentially increased in SCWL sugarcane, acting as antioxidants to protect sugarcane from phytoplasma infection.

Flavonoids and isoflavonoids act as antioxidants to protect plants from pathogens by boosting growth and development [90]. The downregulation of *CHS* and *CYP75B1*, which are key genes of flavonoid biosynthesis, in SCWL sugarcane suggests a reduction in flavonoid accumulation, which, in turn, could contribute to an escalation in disease symptoms when the plant is exposed to a pathogenic challenge [91]. Genes related to diterpenoid biosynthesis in phytoplasma-infected lime were upregulated including *GA2*, *GA3*, *KAO*, *GA2ox*, and *GA20ox* [53] like those found in SCWL sugarcanes in this study. Diterpenoids exhibit a species-specific chemical defense against pathogens [92]. A common precursor of diterpenoids, geranylgeranyl diphosphate, also contributes to GA biosynthesis [93]. Moreover, upregulation of sesquiterpenoid and triterpenoid biosynthesis in SCWL sugarcane may increase β-caryophyllene, δ-elemene, and germacrene D, similarly to those phytochemicals found in phytoplasma-infected *Hypericum perforatum* L. [94]. The increase in sesquiterpene biosynthesis in SCWL sugarcanes may attract the insect vector to disperse SCWL phytoplasma as suggested for AP phytoplasma-infected apple and tobacco [95]. Hence, changes in the biosynthesis of flavonoids, terpenoids, and sesquiterpenes in SCWL sugarcane may constitute a component of the pathogen’s strategy for disease dissemination.

## 4. Conclusions

SCWL sugarcanes at the maturation phase harbor 10^4^ times more phytoplasma than asymptomatic plants growing in the same field and have smaller stalk diameters, shorter height, and lower sugar content. Transcriptomic analysis revealed alterations in gene expression for several pathways in response to an infection by phytoplasma including photosynthesis, porphyrin and chlorophyll metabolism, starch and sucrose metabolism, plant hormone signaling transduction, flavonoid biosynthesis, and plant–pathogen interactions. Suppression of porphyrin and chlorophyll metabolism coupled with an increase in chlorophyllase expression led to a reduction in chlorophyll levels and in photosynthesis that corresponded to the white leaf phenotype. Enhancement of sugar transporters in leaves, but suppression in stalks, suggests that sucrose transport is blocked and results in lower sucrose levels in stalks. Upregulation of *SUS* leads to a UDP-glucose-induced callose plug formation in sieve pores that serves as a defense mechanism against colonization by phytoplasma. The expression of genes associated with calcium cascades led to Ca^2+^ accumulation, which contributed to plant immunity. However, downregulation of *CPK* would result in a reduction in SOD, POD, and CAT activities, consequently facilitating an invasion by the pathogen. Furthermore, enrichment of signaling transduction pathways (e.g., *MAPK*, *CAT*, *PR1*, *GK*, and transcription factors) and plant hormone signaling transduction (e.g., ABA, AUX, CK, GA, JA, SA, and ETH-responsive genes) positively promotes the defense response of sugarcane against phytoplasma. It is speculated that phytoplasma secretion effectors might target TCPs and cause an expression of *LOX* and subsequently increase the JA level. Upregulation of the phenylpropanoid pathway led to lignin accumulation in response to an attack by phytoplasma, while the expression of sesquiterpene biosynthesis possibly attracts the insect vectors for transmission, thereby facilitating the spread of phytoplasma to other sugarcanes. However, downregulation of flavonoid biosynthesis would potentially intensify the SCWL symptoms upon challenge by phytoplasma. These SCWL sugarcane transcriptomic profiles represent the first comprehensive view of genes and pathways involved in the response of sugarcane towards infection by phytoplasma, which will facilitate in finding specific corresponding marker genes for further sustainable development of disease prevention, protection, and management.

## 5. Materials and Methods

### 5.1. Sugarcane Samples and Plant Growth Parameters

*Saccharum* hybrid cv. KK3 was grown in a field of the MitrPhol sugarcane plantation, Nong Kung Si district, Kalasin province, Thailand (16°42′56.0″ N 103°22′34.2″ E). Treatments of organic fertilizers in January and chemical fertilizers in February [N-P-K = 21-7-18, 312.5 kg/ha April (N-P-K = 46-0-0, 156.25 kg/ha and 21-7-18, 156.25 kg/ha), and July (N-P-K = 21-7-18, 312.5 kg/ha)] were applied. Five of the 12-month-old symptomatic and asymptomatic SCWL sugarcanes were randomly harvested from the same location. Phenotypic characteristics of SCWL sugarcanes were determined visually based on the basic paler leaves compared to the asymptomatic ones. Sugarcane stalks of the 14th–16th internode from the root and 1st–3rd leaves from shoots were immediately sampled between 11 a.m. and 4 p.m. and kept in an RNA storage reagent (Tiangen, Beijing, China) at −20 °C until use. Plant growth parameters including height and stalk diameter were measured. Sugar content was analyzed using a master refractometer (ATAGO, Tokyo, Japan) and recorded as a Brix percentage.

### 5.2. RNA Extraction

Both stalk and leaf samples were cut into small pieces and immediately frozen with liquid nitrogen and ground to a fine powder. Total RNA was extracted using a GF-1 total RNA extraction kit (Vivantis, Shah Alam, Malaysia), as described by the manufacturer. RNA quality and quantity were analyzed using a Nanodrop ND-100 (Thermo Scientific, Waltham, MA, USA).

### 5.3. Detection of Phytoplasma in Sugarcane Using Real-Time PCR

DNA samples of SCWL sugarcane were prepared using the CTAB method [96]. The phytoplasma 16S rRNA gene was amplified by nested PCR using primers described previously [48]. The PCR master mix contained 100 ng DNA template, 1× Phusion HF buffer, 200 µM dTNPs, 0.5 µM each primer, and Phusion Hot Start II DNA polymerase (0.02 U/µL) (Thermo Scientific, Waltham, MA, USA). Sugarcane 18S rRNA gene was amplified using 18S rRNA-F and 18S rRNA-R primers [97] following the PCR reaction above. The purified PCR products were sent for sequencing at Macrogen (Seoul, Republic of Korea). The numbers of phytoplasma 16S rRNA and sugarcane 18S rRNA genes were calculated using the formula of Staroscik [98] to generate standard curves.

To determine the number of active phytoplasma in sugarcanes, total RNA was converted to cDNA using random hexamers and RevertAid First strand cDNA synthesis (Thermo Scientific, Waltham, MA, USA). Real-time PCR was performed using a Master Cycler Realplex 4 (Eppendorf, Hauppauge, NY, USA) with a KAPA SYBR FAST qPCR master mix (Kapa Biosystems, Seoul, Republic of Korea) with reaction conditions: 95 °C for 3 min, 40 cycles at 95 °C for 10 s, and 62 °C for 1 min. Quantification of 16S rRNA genes of phytoplasma and 18S rRNA genes of sugarcane was calculated based on the standard curve using comparative cycle threshold (Ct) values [99]. The content of phytoplasma in each sample was normalized using the corresponding sugarcane 18S rRNA gene.

### 5.4. RNA Sequencing and Transcriptome Analysis

Total RNA samples of 3 biological replicates of symptomatic SCWL leaves (SL), asymptomatic leaves (AL), symptomatic SCWL stalks (SS), and asymptomatic stalks (AS) were sent for RNA sequencing at NovogeneAIT Genomics Singapore Pte (Singapore). Briefly, RNA samples with an RNA integrity number (RIN) of more than 6.5 were subjected to rRNA depletion, mRNA random fragmentation, cDNA construction, and terminal end ligation with poly-A and sequencing adaptors. Then, cDNA libraries were size selected and quantified before sequencing using an Illumina Hiseq X instrument (2 × 150 bp) (Illumina, San Diego, CA, USA).

Raw reads were uploaded to Galaxy Project (usegalaxy.org) and initially were quality checked using FastQC [100]. Low-quality sequences at Q20 and adapter contamination were filtered out using Trimmomatic [101]. The clean reads were mapped to the monoploid sugarcane genome reference (https://sugarcane-genome.cirad.fr; accessed on 14 February 2020) using Bowtie2/TopHat [102]. The mapped reads were assembled using Cufflink, merged using Cuffmerge, and performed DEGs using Cuffdiff [103]. Principal component analysis (PCA) was performed using DESeq2 [104].

DEGs were cut off at log_2_ FC ≥ 1 with a false discovery rate (FDR) of <0.05. Gene function was categorized using gene id and protein products from the gene ontology (GO) [11] and general feature format (GFF) annotation file provided by the reference genome sequence [11]. GO terms of all DEGs were subjected to singular enrichment analysis (SEA) in agriGO [105]. The GO term enrichment was calculated by comparing the frequency of gene sets with different GO terms relative to reference gene sets of Plant GO slim using Fisher as statistical test method, Benjamini–Yekutieli as a multi-test adjustment method, and 5 as a minimum number of mapping entries [106]. Then, biological pathways and molecular interaction networks of DEGs were classified by mapping amino acid sequences using GhostKOALA and KEGG mapper [107,108]. Pathway enrichment analysis was performed using the piano in R package based on geneSetStat “reporter” [109]. Pathways with *p* values of <0.05 were considered as significantly enriched pathways.

### 5.5. Quantification of Candidate Genes by Real-Time PCR

One microgram of total RNA was used for cDNA synthesis with oligodT primer using RevertAid First strand cDNA synthesis (Thermo Scientific, Waltham, MA, USA). The candidate genes were randomly selected from the significantly enriched pathways and primers were accordingly designed using Primer BLAST [110] (Table S8). Real-time PCR was performed in 3 biological and 3 technical replicates using a Master Cycler Realplex 4 (Eppendorf, Hauppauge, NY, USA) with KAPA SYBR FAST qPCR master mix (Kapa Biosystems, Seoul, Republic of Korea) using reaction conditions: 95 °C for 3 min, 40 cycles at 95 °C for 10 s, and 59 °C for 1 min. Relative levels of the expression of DEGs of SCWL and asymptomatic sugarcanes were determined using the ΔΔC_T_ method [111] with the *GAPDH* gene used as an internal control for data normalization.

### 5.6. Statistical Analysis

Plant growth parameters were analyzed by *t*-test to determine statistical significance between groups at *p*-values of <0.05 by Microsoft Excel data analysis.

## Figures and Tables

**Table 1:** Growth parameters, sugar content, and phytoplasma ratio of asymptomatic and symptomatic sugarcanes.

Sugarcane	Height (cm)	Diameter (cm)	Sugar Content (°Brix)	16S/18S rRNA Gene Ratio	
				Leaves	Stalks
Asymptom	302.20 ± 12.77	3.34 ± 0.13	22.84 ± 0.25	2.35 × 10^−5^ ± 0.82 × 10^−5^	8.96 × 10^−5^ ± 3.00 × 10^−5^
Symptom	199.20 ± 16.76 *	2.18 ± 0.16 *	15.86 ± 1.14 *	0.33 ± 0.10 *	0.57 ± 0.14 *

Asterisk (*) indicates significant differences by unpaired *t*-test analysis.

**Table 2:** Number of DEGs in gene ontology (GO) and KEGG pathway in SCWL leaves and stalks.

Category	Number of DEGs	
	Leaves	Stalks
Significantly expressed gene (*p*-value < 0.05)	6612	2173
|log_2_Fold Change| > 1.0	6284	1737
- Upregulated genes	4799	994
- Downregulate genes	1485	743
Gene ontology	3711 (59.06%)	987 (56.82%)
- Molecular function	3350	901
- Cellular component	884	186
- Biological process	2523	675
KEGG pathway	2248 (35.79%)	531 (30.57%)
- Upregulated DEGs	1701	294
- Downregulated DEGs	547	237

## Data Availability

RNA sequences are deposited to GenBank database as a sequence read archive (SRA) accession number SRR14169813-24 in the BioProject number PRJNA719388.

## Supplementary Materials

The following supporting information can be downloaded at: https://www.mdpi.com/article/10.3390/plants13111551/s1. Figure S1: Effect of phytoplasma on sugarcane growth and physiology; Figure S2: Volcano plots of upregulated and downregulated DEGs in comparison between SCWL and asymptomatic sugarcane leaves and stalks; Figure S3: Gene ontology enrichment analysis of DEGs under phytoplasma infection in sugarcane leaves and stalks; Table S1: Quality assessment of RNA-seq data; Table S2: List of enrichment pathways in leaves; Table S3: List of enrichment pathways in stalks; Table S4: List of differentially expressed genes (DEGs) within the targeted pathway in leaves; Table S5: List of differentially expressed genes (DEGs) within the targeted pathway in stalks; Table S6: List of transcription factors in leaves; Table S7: List of transcription factors in stalks; Table S8: List of candidate genes and primers used for real-time PCR in this study.
