# Supplementary material for: Transcriptomic Profiling of Sugarcane White Leaf (SCWL) Canes during Maturation Phase
Source: Plants (Basel). 2024 Jun 4;13(11):1551. doi: 10.3390/plants13111551 (PMC11174868; doi:10.3390/plants13111551)
Supplement: Supplementary file 1 [file plants-13-01551-s001.zip › Lomaneeratana et al_Plants_Supplement Tables and Figures.pdf]

Suplementary Materials

Transcriptome profiling of sugarcane white leaf (SCWL) canes during maturation and ripening phase

Karan Lohmaneeratana[1], Kantinan Leetanasaksakul[2], Arinthip Thamchaipenet[1,3]*

[1]Department of Genetics, Faculty of Science, Kasetsart University, Bangkok10900, Thailand

[2]National Center for Genetic Engineering and Biotechnology, National Science and Technology Development Agency, Pathumthani 12120, Thailand

[3]Omics Center for Agriculture, Bioresources, Food and Health, Kasetsart University (OmiKU),

Bangkok 10900, Thailand

*Corresponding author

E-mail address: arinthip.t@ku.ac.th (A. Thamchaipenet)

Table S1 Quality assessment of RNA-seq data

Samples	Replicates	Raw reads	Clean reads	Q20 (%)	GC Content (%)	Mapping read (percentage)
Leaf	AL1	57,085,553	56,427,208	96	51.64	45,167,555 (80.05%)
	AL2	57,437,146	56,836,916	96.07	51.91	45,842,826 (80.66%)
	AL3	57,944,354	57,292,421	96.03	51.34	44,897,906 (78.37%)
	SL1	54,549,153	54,060,606	96.91	51.58	41,219,902 (76.25%)
	SL2	54,626,924	54,108,590	96.82	50.5	37,593,049 (69.48%)
	SL3	54,586,266	53,866,262	95.73	50.99	39,762,992 (73.82%)
Stalk	AS1	54,983,573	52,818,601	97.1	52.41	41,647,466 (78.85%)
	AS2	55,772,825	53,251,981	96.91	51.42	41,935,935 (78.75%)
	AS3	55,382,450	52,934,151	96.92	52.28	42,013,835 (79.37%)
	SS1	59,838,759	56,188,488	95.78	52.12	42,484,115 (75.61%)
	SS2	55,266,485	53,005,954	97.09	51.19	39,961,188 (75.39%)
	SS3	53,864,118	51,552,491	96.96	52.08	40,808,951 (79.16%)

Table S8 List of candidate genes and primers used for real-time PCR in this study

Genes	Gene ID	Primers Sequences (5'-3')	Tm (°C)	Size (bp)
**Leaf**				
Auxin-responsive protein SAUR21 (*SAUR*)	Sh_220G15_p000040	F: CCTCATCGGCCAGGACCTC<br>R: GTGCGCGGGTCCTCCTC	60	100
Catalase isozyme 3 (*CAT*)	Sh_019K12_p000030	F: CAGGAGAGGTTCGTCAGAAGG<br>R: GATTTACCTTGGAGAGGAGGTCG	60	100
Basic endochitinase C (*ChiB*)	Sh_237K21_p000020	F: CTGGTCCTGGCAGGCG<br>R: GGCTCAGCATGCTCTGGAA	60	109
Transcription factor WRKY33 (*WRKY33*)	Sh_242F09_p000080	F: CAGCAGCAGTCGCAGAGG<br>R: CGCTCCACCTTCTTCTTGGT	60	143
Rac-like GTP-binding protein 4 (*RAC*)	Sh_245M05_p000020	F: ACTCCAGCAGACGAGACGA<br>R: TTTGGTGTTGTTGTGGCCTT	60	103
Fructose-1,6-bisphosphatase (*FBP*)	Sh_235E04_contig-1_p000090	F: CCAGTAGCGGTGGAACAGAG<br>R: GCGTAGATGCCGAAGATGGA	60	116
Trehalose 6-phosphate phosphatase (*OtsB*)	Sh_225I16_p000080	F: GTGTACATCGGCGACGACC<br>R: ATCACTGACCTCGGCGG	60	150
Chloroplast photosystem II 22 kDa protein (*PsbS*)	Sh_002A11_p000060	F: CAAGCCGAAGGTCGAGGATG<br>R: CACGAGTAATCACGGCAAAGC	60	116
Chlorophyll a-b binding protein (*Lhca2*)	Sh_222O22_p000140	F: CCACTGCAACATCTTCTCCGT<br>R: ACTCCGAAACAGCATCGTATC	60	100
Chlorophyllase-2 (*Chlorophyllase*)	Sh_205M16_p000090	F: GCCGCTCAACATGAAAGACC<br>R: GGTGGAGGATGTTGGTGAGG	60	110
Flavonoid 3' monooxygenase (*CYP75B1*)	Sh_235P15_p000020	F: ATGGCGTACAACTACCAGGAC<br>R: ACCGCCGGCGCATTATT	60	197
flavonoid 3',5'-hydroxylase (*CYP75A*)	Sh_223L22_p000060	F: CGGGCACGGACACATCTT<br>R: CAAGTAAGGAAGGTTGGGCA	60	155
Indole-3-glycerol phosphate lyase (*TrpA*)	Sh_241K17_contig-2_p000020	F: GGCGGAGGTCTGTAGGCA<br>R: ACACCGTCAGGCATCGGC	61	105
3'-phosphoadenosine 5'-phosphosulfate synthase (*PAPSS*)	Sh_229P05_p000030	F: AAGAAAGAATCGCAAGAACATTCG<br>R: AGGTCACCACCGATCAGC	59	100
**Stalk**				
Transcription factor WRKY24 (*WRKY24*)	Sh_027M21_p000160	F: CAGCGCCGCACTCTACC<br>R: GGCGCCGAACCCGTA	60	127
Alcohol dehydrogenase2 (*ADH1*)	Sh_210D04_p000020	F: TGTGGGTCTTGCTGCTATGG<br>R: TGGCTTGTCATAGTCCTTGGG	60	142
Calcium-binding protein CML (*CML*)	Sh_239L16_p000030	F: GTCGGATCTCGCGGTCG<br>R: CGAACTCGTCCAGGCTGATG	60	132
Phenylalanine ammonia-lyase (*PAL*)	Sh_206K06_p000070	F: GACCACGAGCCTGGACGG<br>R: GCCCGGATCTTCTTCATCAGG	60	141
Aldose reductase (*adh*)	Sh_250M05_p000060	F: AAGAACCTAGCACACGACCC<br>R: AACGCTTGTTCCCCTTTGGA	60	105
Pyruvate, orthophosphate dikinase (*ppdK*)	Sh_250G13_p000060	F: CGCAGGTGCCGTAAAGATTG<br>R: GTAGGCCCGTGGCAATAACT	60	161
**House-keeping gene**				
Glyceraldehyde-3-phosphate dehydrogenase 3 (*GAPDH*)	Sh_251N21_p000050	F: GGTGCCGTTGGTGCTGAATA<br>R: CACCAACAACGAACATGGGG	60	142

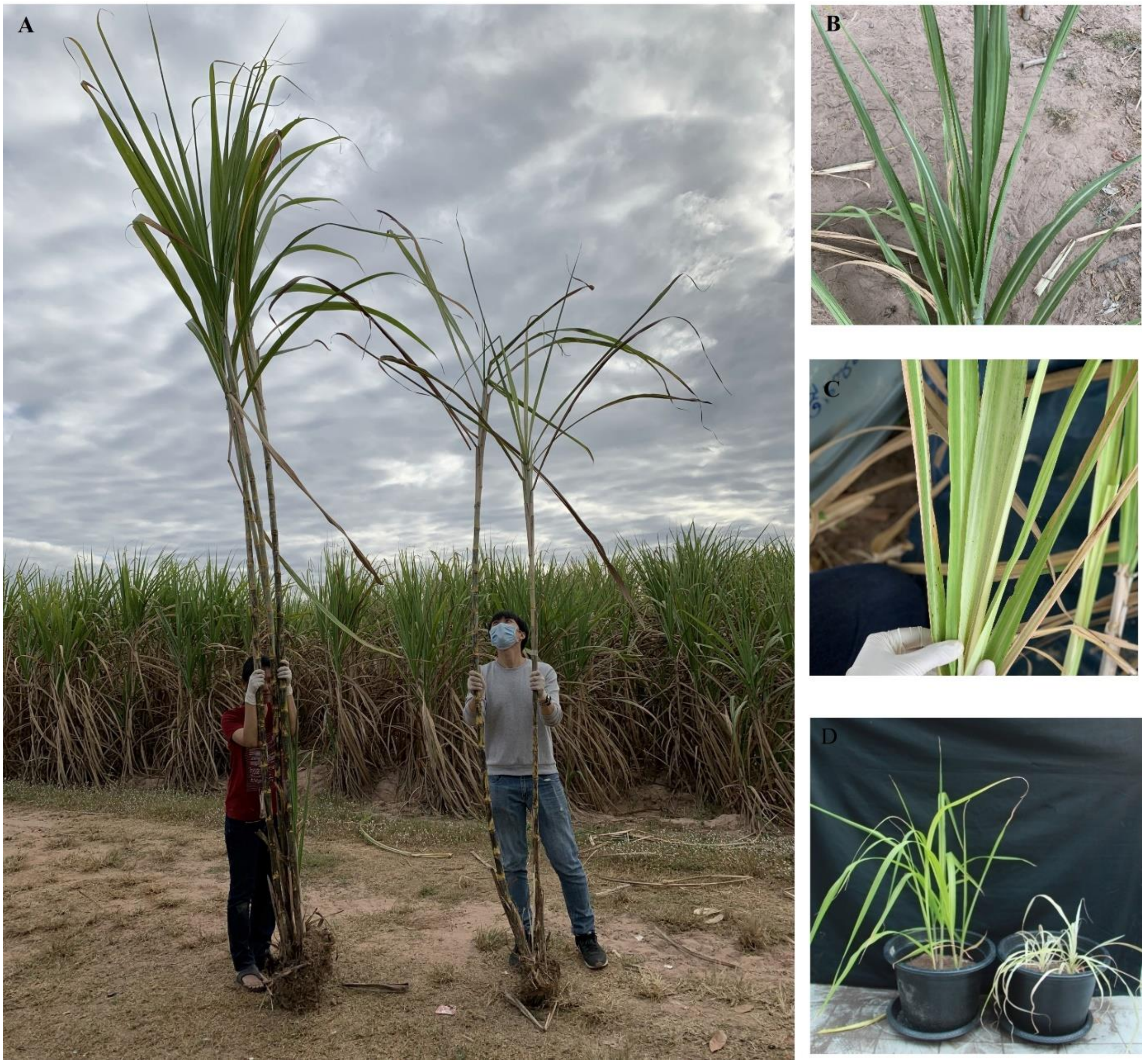

Figure S1 Effect of phytoplasma on sugarcane growth and physiology. (A) Twelve-month-old asymptomatic (left) and symptomatic SCWL (right) sugarcanes growing in the same plantation, (B) asymptomatic leaves, (C) SCWL leaves, (D) two-month-old asymptomatic (left) and symptomatic SCWL (right) derived from the corresponding seed canes.

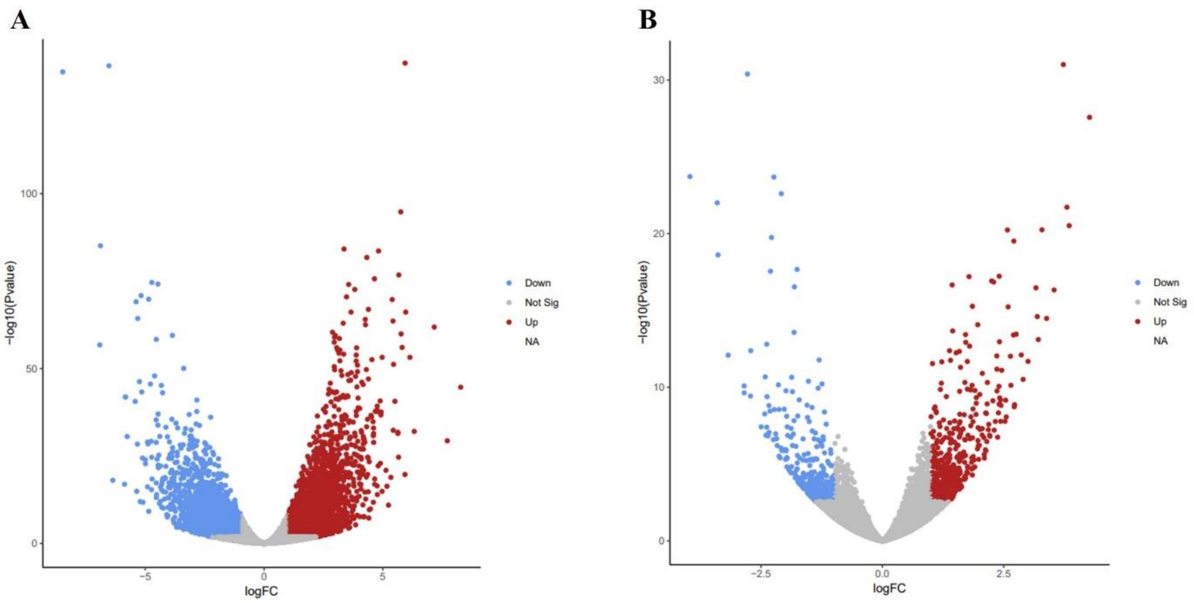

Figure S2 Volcano plots of up- and downregulated DEGs (red and blue dots, respectively) in comparison between SCWL and asymptomatic sugarcane leaves (A) and stalks (B).

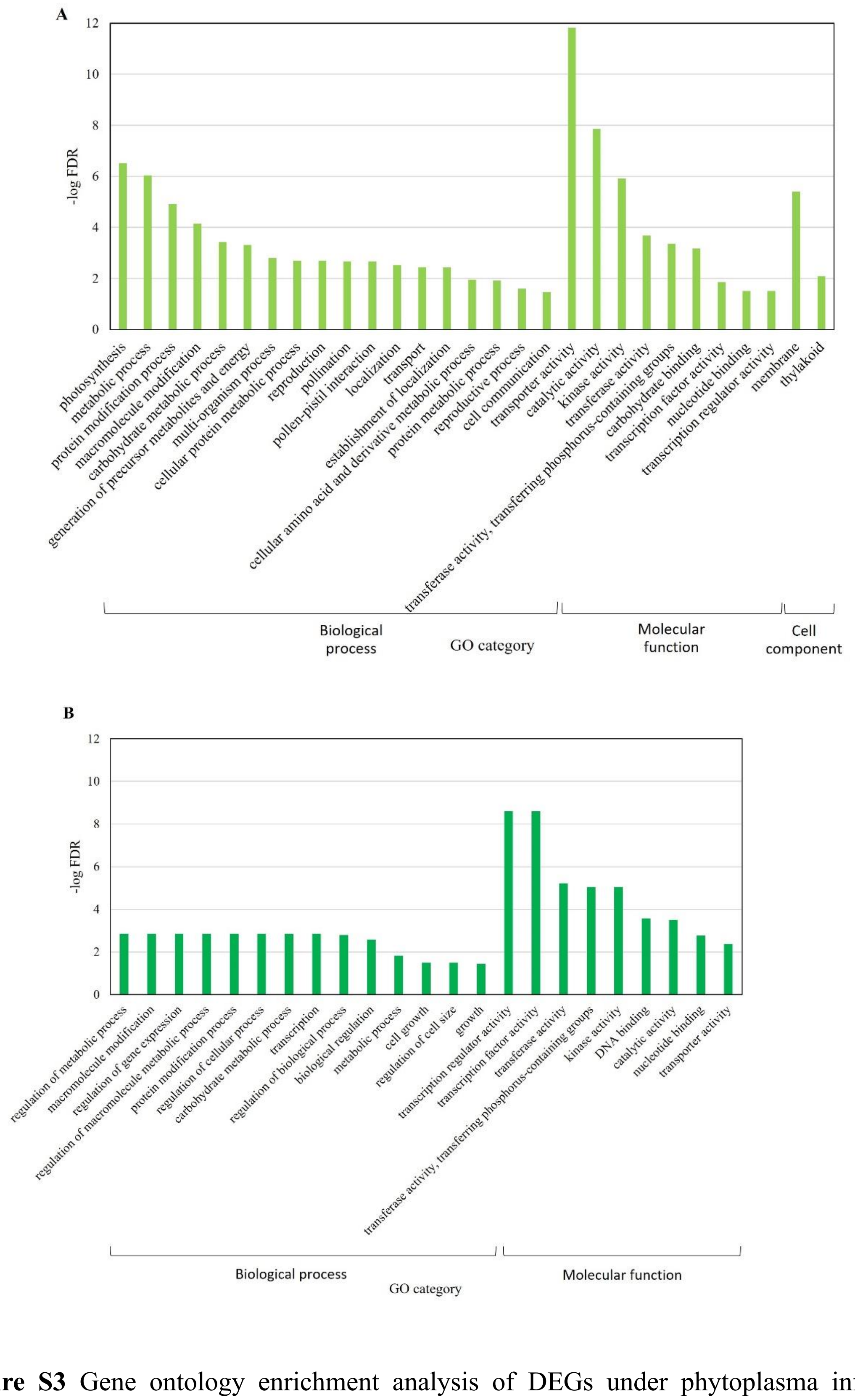

Figure S3 Gene ontology enrichment analysis of DEGs under phytoplasma infection in sugarcane leaves (A) and stalks (B). GO biological process, cellular component, and molecular function enrichment analyses were performed using Fisher method. Significantly enriched GO terms are supported by Benjamini-Yekutieli FDR-corrected *p*-values.
